# Legitimate Dental Practice Hampered by Law

**Published:** 1898-12

**Authors:** 


### Domestic Correspondence.

### LEGITIMATE DENTAL PRACTICE HAMPERED BY LAW.

To the Editor:

Sir,—In a paper read before the New Jersey State Dental Society, at Asbury Park, July, 1898, Dr. William I. Wallace, of Glens Falls, N. Y., read a paper entitled "Experience with a Few Homœopathic Remedies in Dental Practice."[1] In this paper Dr. Wallace recommends a few doses of aconite, the third potency, about half an hour apart, in those cases of neuralgia of the fifth nerve, which are frequently reported to the dentist under the impression that a tooth is in fault. He also recommends a few doses of belladonna, third potency, for relief of pulpitis in its earlier stages; mercurius corr. sub. 30, a dose every hour until better, in neglected cases of inflammation, where there is evidence of pus forming; chamomilla 30, in toothache of children when the pain is due to caries, as a temporary relief, creosotum administered internally to retard certain forms of caries, and silicea as a tonic, in alveolar abscess of slow development and tardy recovery. The above gives all the suggestions directly made in the paper, and are here given that Dr. Brown's answer to Dr Watkins's question may be understood, and its import fully appreciated. It should be noted that Dr. Wallace does not suggest a dentist branching out into general medicine. Although before entering the dental profession Dr. Wallace practised medicine, and this prior experience led to the use of these remedies, he has not, in his paper, been led by this beyond the strict limits of dental practice. All these cases, with a possible exception to the first, are purely dental, and legitimately belong to a dentist's care.

During the discussion that followed the reading of the paper the propriety of a dentist administering internal remedies, and his legal right to do so when, in his judgment, the best interests of his patient requires it, was questioned. Regarding the legal right, so far as New Jersey is concerned, and probably other States having similar laws, the following question and answer is of interest on this point.

---

[1] Items of Interest, September, 1898, p. 668.

"*Dr. S. C. G. Watkins.*—I would like to ask Dr. Brown if our law does not give us the privilege of prescribing medicine for dental troubles?

"*Dr. Brown.*—I doubt it; we have had one or two opinions on that, and the line is so close that it never can be settled until it goes to the Supreme Court. But I doubt very much if the courts would uphold a dentist who administered medicine in the manner suggested in this paper."—*Items of Interest*, September, 1898, p. 687.

Dr. Brown's long and active service as a leader in shaping and enforcing dental law in New Jersey gives force and weight to this expression of opinion. . . . But what has he and his associates been about to permit such a state of affairs? Their dental law, if I mistake not, expressly permits a physician to extract teeth, and to perform quite a variety of dental operations; indeed, outside of the purely mechanical operations of dental art. What may not a physician do, as a physician, to relieve a patient suffering from dental troubles? If it is, and has been, as Dr. Brown says, a disputed question whether a dentist, as a dentist, may or may not legally do all that his judgment directs to relieve a patient suffering from trouble that properly comes under his care, it is so because those who have framed the laws regulating the practice of dentistry have so permitted it.

